# Correction: Tsai et al. The Efficacy of Transvaginal Ultrasound-Guided BoNT-A External Sphincter Injection in Female Patients with Underactive Bladder. *Toxins* 2023, *15*, 199

**DOI:** 10.3390/toxins15060399

**Published:** 2023-06-16

**Authors:** Cheng-Yen Tsai, Yao-Hung Yeh, Li-Hsien Tsai, Eric Chieh-Lung Chou

**Affiliations:** 1 Department of Urology, China Medical University Hospital, Taichung 40447, Taiwan; cytsai1611@gmail.com; 2 School of Medicine, China Medical University, Taichung 40402, Taiwan; 3 Department of Radiology, Kaohsiung Veterans General Hospital, Kaohsiung 81362, Taiwan; yhyeh2019@gmail.com; 4 School of Medicine, Chung Shan Medical University, Taichung 40201, Taiwan

## Title Mistake

In the original publication [1], there was a mistake in Section 3. During the first major revision, the authors used ChatGPT to perform the English editing for the newly added content. However, they neglected to remove the phrase “regenerate response” in their haste. Now, the authors request to correct this error and remove this title from the article.
